# CRISPR/Cas9‐mediated editing of *Bs5* and *Bs5L* in tomato leads to resistance against *Xanthomonas*

**DOI:** 10.1111/pbi.14404

**Published:** 2024-07-12

**Authors:** Arturo Ortega, Kyungyong Seong, Alex Schultink, Daniela Paula de Toledo Thomazella, Eunyoung Seo, Elaine Zhang, Julie Pham, Myeong‐Je Cho, Douglas Dahlbeck, Jacqueline Warren, Gerald V. Minsavage, Jeffrey B. Jones, Edgar Sierra‐Orozco, Samuel F. Hutton, Brian Staskawicz

**Affiliations:** ^1^ Department of Molecular and Cell Biology University of California, Berkeley Berkeley California USA; ^2^ Department of Plant and Microbial Biology University of California, Berkeley Berkeley California USA; ^3^ Innovative Genomics Institute, University of California, Berkeley Berkeley California USA; ^4^ Fortiphyte Inc. Richmond California USA; ^5^ Department of Plant Pathology University of Florida Gainesville Florida USA; ^6^ Gulf Coast Research and Education Center University of Florida Wimauma Florida USA

**Keywords:** Bs5, disease resistance, CRISPR/Cas9 technology, bacterial spot disease, crop engineering

Bacterial spot, caused by *Xanthomonas* species, is a devastating disease of tomato (*Solanum lycopersicum*) and pepper (*Capsicum annuum*) (Schwartz *et al*., 2015). The recessively inherited resistance, *bacterial spot 5* (*bs5*), in pepper (hereafter referred to as *Cabs5*) can confer resistance against different *Xanthomonas* strains (Jones *et al*., 2002). The *Cabs5* resistance is characterized by the absence of disease symptoms, faint chlorosis at the site of infection, and reduced bacterial growth. Remarkably, commercial pepper varieties containing the *bs5* allele show durable resistance, effectively impeding hypervirulent strain emergence in agricultural fields (Vallejos *et al*., 2010).

The *CaBs5* gene, together with its paralog *CaBs5‐like* (*CaBs5L*), has recently been cloned (Sharma *et al*., 2023; Szabó *et al*., 2023). *CaBs5* encodes a 92 amino acid long protein possessing a cysteine‐rich transmembrane (CYSTM) domain, which is implicated in various biotic and abiotic responses. Typically, the CYSTM domain contains conserved residues composed of four consecutive cysteines, followed by two hydrophobic amino acids. A recent study suggested that Cabs5 mediating the resistance against bacterial spot lacks these two conserved leucine residues within the CYSTM domain (Szabó *et al*., 2023).

Tomatoes and peppers are close relatives in the Solanaceae family and commonly susceptible to *Xanthomonas* infection. Based on the current findings in pepper, we hypothesized that modifying the ortholog of *CaBs5* in tomato could confer resistance against *Xanthomonas*. Consequently, putative *Bs5* (*SlBs5*) and *Bs5L* (*SlBs5L*) were identified in tomato based on homology to *CaBs5*. Both *SlBs5* and *SlBs5L* were located on chromosome 9 with the same head‐to‐head orientation as their pepper homologues on chromosome 3 (Figure 1a). Despite short and highly similar amino acid sequences of SlBs5 and SlBs5L (Figure 1b), the conserved synteny and gene order in pepper and tomato genomes allowed the assignment of orthology for *Bs5* and *Bs5L*.

**Figure 1:**
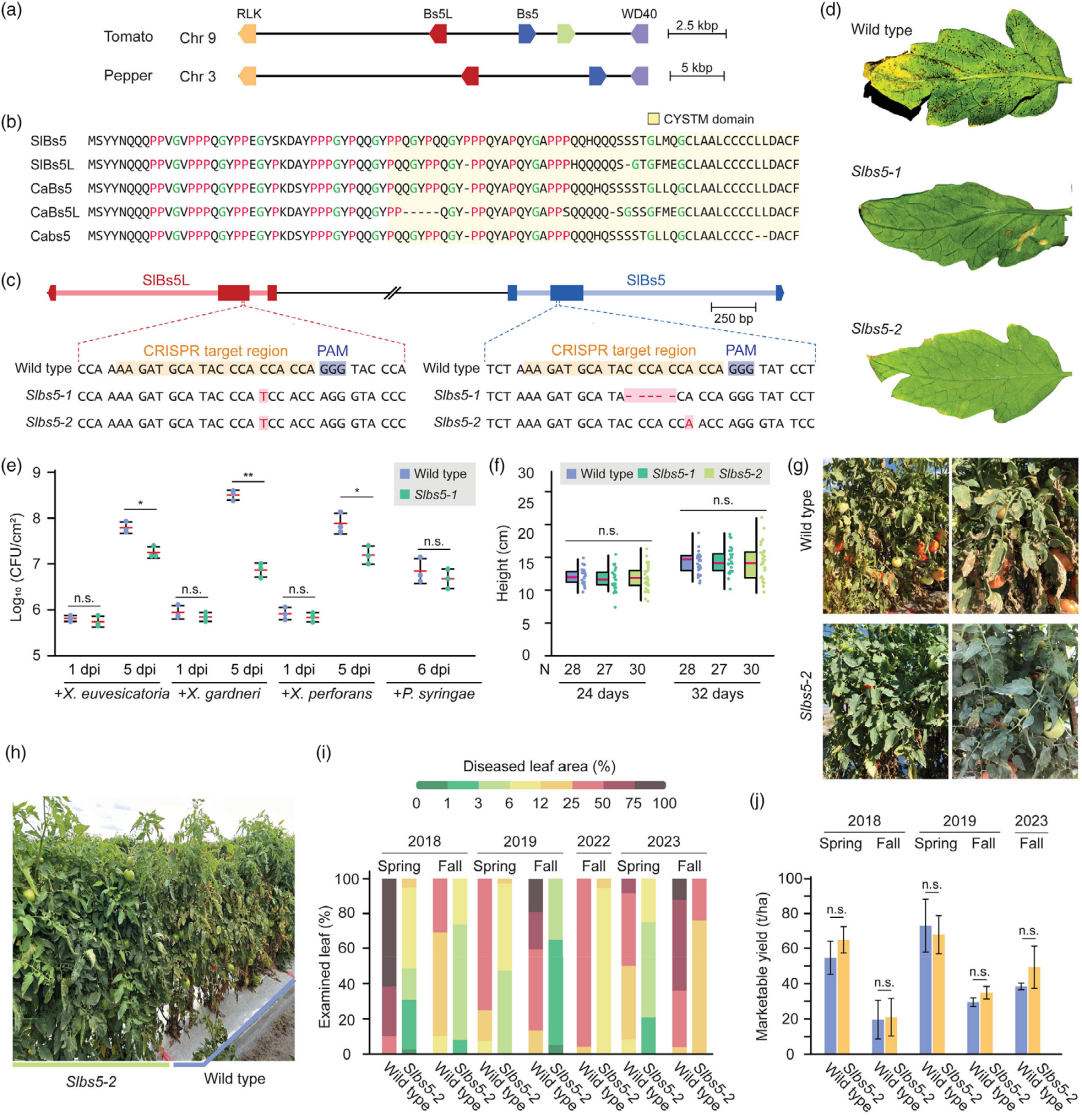
CRISPR/Cas9‐mediated editing of *Bs5* and *Bs5L* in tomato leads to resistance against *Xanthomonas* in laboratory and field conditions. (a) The genomic architecture around tomato (*Solanum lycopersicum*) and pepper (*Capsicum annuum*) *Bs5* and *Bs5‐Like* (Bs5L) commonly flanked by a leucine‐rich receptor‐like kinase (LRR‐RLK) and a WD40 transcription factor. (b) The sequence alignment of *S. lycopersicum* Bs5 and Bs5L (SlBs5 and SlBs5L) and *C. annuum* Bs5, Bs5L and bs5 (CaBs5, CaBs5L and Cabs5). (c) CRISPR/Cas9 target design and resulting genotypes. (d) Qualitative evaluation of disease symptoms against X*anthomonas perforans* GE485 at 21 days post‐inoculation. (e) Quantitative evaluation of bacterial growth in the given days post‐infiltrations (dpi). Statistical significance was determined with two‐tailed pairwise *t*‐tests with the Benjamini–Hochberg procedure (n.s. *P* ≥ 0.05; *P < 0.05; **P < 0.01). (f) The heights of plants grown in the laboratory conditions with given numbers of replicates (N). Statistical significance was determined with two‐tailed pairwise *t*‐tests with the Benjamini–Hochberg procedure (n.s. *P* ≥ 0.05). (g, h) The wild‐type and *Slbs5‐2* plants in the field trial during Fall 2019 and Fall 2023, respectively. (i) The area of leaves impacted by bacterial spot disease categorized based on the given scale for the wild‐type and *Slbs5‐2* plants. (j) Marketable fruit yields of wild‐type and *Slbs5‐2* plants. Two‐tailed pairwise *t*‐tests were performed with the Benjamini–Hochberg procedure (n.s. *P* ≥ 0.05).

The mechanism by which the double leucine deletion in *Cabs5* leads to resistance against *Xanthomonas* remains elusive (Figure 1b). Yet, this deletion in the conserved CYSTM domain could potentially impair CaBs5's native functionality (Abell and Mullen, 2011). Following this assumption, we postulated that knocking out *SlBs5* would produce similar outcomes to *Cabs5*. We aimed to disrupt both SlBs5 and SlBs5L to prevent possible functional complementation by SlBs5L, given their greater amino acid sequence similarity compared to CaBs5 and CaBs5L (Figure 1b).

We constructed a binary vector for Cas9 and a single‐guide RNA (sgRNA) targeting conserved sequences present in both *SlBs5* and *SlBs5L* (Figure 1c). Tomato variety Fla. 8000 was transformed with *Agrobacterium*. From the progeny of successful transformants, we selected two homozygous lines, *Slbs5‐1* and *Slbs5‐2*, containing frameshift mutations in both genes (Figure 1c). These mutant lines were self‐pollinated or backcrossed to the wild‐type parent variety to segregate the T‐DNA containing the Cas9‐sgRNA cassette.

The resistance of the two selected mutant lines was qualitatively evaluated against *Xanthomonas perforans* GE485 with dip inoculation assays (Figure 1d). At 21 days post‐inoculation, the wild‐type leaves were covered by black spots indicative of *Xanthomonas* infection, while both *Slbs5‐1* and *Slbs5‐2* retained green leaves with fewer visible symptoms. These phenotypes remained consistent in inoculations of *X. perforans* 4B and *Xanthomonas gardneri* 153 (Figure S1).

Quantitative evaluation of bacterial growth further supported these findings. At 5 days post‐infiltration with a low‐density bacterial suspension, *Slbs5‐1* showed significant decreases in *Xanthomonas* populations compared to wild‐type plants (Figure 1e). Such reductions were consistently observed for *Slbs5‐2* (Figure S2). However, *Slbs5‐1* could not significantly hinder *Pseudomonas* population growth.

We additionally examined the growth penalty associated with *Slbs5‐1* and *Slbs5‐2* in controlled conditions (Figure 1f). The height of plants was measured at two different time points, but no significant differences were observed between the wild type and the two mutant lines (Figure 1f; Figure S3). This suggested that the resistance to *Xanthomonas* species comes at no developmental cost in the vegetative stage in the laboratory setting.

Although *Cabs5*‐mediated immunity is subtle, it has shown practical value in commercial pepper cultivation. To examine the commercial potential of *Slbs5*, field trials were conducted with both *Slbs5‐1* and *Slbs5‐2* lines at the Gulf Coast Research and Education Center in Florida, a major state for tomato production. Along with naturally occurring *Xanthomonas* populations, a two‐isolate cocktail of *X. perforans* race T4 was inoculated in the field to heighten disease pressure. Plants were grown with recommended fertilizers and pest management programs, excluding the use of any bactericides or activators of systemic acquired resistance.

Despite seasonal variations, *Slbs5* mutant lines consistently maintained reduced disease symptoms (Figure 1g). Additionally, no developmental defects, such as stunting, were observed in these mutants (Figure 1h). Quantification of disease severity, based on visible symptoms caused by *Xanthomonas* infection on plant leaf surfaces, revealed higher percentages of *Slbs5‐2* leaves with reduced disease symptoms than wild‐type leaves in all tested seasons (Figure 1i; Figure S4). Notably, the *Slbs5‐2* mutants demonstrated effective resistance during three periods of elevated disease pressure, Spring 2018, Fall 2019, and Fall 2023.

The marketable yield of fruits is a critical consideration in tomato cultivation. We quantified total marketable yield across five seasonal trials, except for two seasons impacted by a hurricane (Fall 2022) and extremely dry weather (Spring 2023). Throughout all seasons, there was no statistically significant difference in marketable fruit yields between *Slbs5‐2* and the wild‐type plants (Figure 1j; Figure S5). However, during the three periods of increased disease prevalence in Spring 2018, Fall 2019, and Fall 2023 (Figure 1i), the mutants consistently showed a tendency to produce a greater quantity of marketable tomatoes (Figure 1j). This possibly suggests a correlation between *Xanthomonas* resistance of the mutant lines and improved fruit yields.

Overall, this study shows that a knockout of *SlbBs5* and *SlBs5L* in tomatoes represents a promising strategy to achieve broad‐spectrum resistance to bacterial spot disease. Compared to stronger sources of resistance, the resistance mediated by *Slbs5* and *Slbs5L* may be considered subtle. However, our mutant lines consistently led to a reduced population of *Xanthomonas* in laboratory and field conditions. This decrease in pathogen populations could lessen the likelihood of hypervirulent strain emergence. Furthermore, when these mutants are combined with other sources of downstream resistance genes, they may serve as a prior layer of defence. This initial protection has the potential to diminish the probability of pathogen effectors directly interacting with and overcoming the resistance genes, possibly extending the efficacy of durable resistance in the agricultural field.

## Author contributions

A.O. and B.J.S. conceptualized the project. B.J.S. supervised the project. A.O., D.D. and B.J.S. designed the experiments and helped analyze the data. K.S. and E.S performed bioinformatics analyses. K.S. led statistical analyses and designed the figures. A.S. helped plan the project, designed and tested the guide RNAs and did preliminary genotyping and bacterial disease assays. A.O. did further genotyping, guide RNA testing and conducted disease and phenotype assays of progeny. D.P.T.T., J.V.W., J.B.J, G.M, E.S.O and D.D. performed supplemental bacterial growth assays. E.S. and S.H. conducted field trials. M.J.C. supervised the generation of tomato mutant lines. E.Z. and J.P. conducted tomato transformations. A.O., K.S. and D.P.T.T. analyzed the data and wrote the manuscript.

## Supporting information


**Appendix S1** Materials and Methods.

**Figure S1** Qualitative evaluation of disease symptoms with a dip inoculation assay.
**Figure S2** Quantitative evaluation of bacterial growth after inoculation.
**Figure S3** Height comparison between wild type and mutant plants.
**Figure S4** The disease symptoms on wild type and Slbs5‐1 plant leaves in the field trials.
**Figure S5** Fruit yields of wild type and Slbs5‐1 plants in the Fall 2023 field trial.

## Data Availability

The data that support the findings of this study are available on request from the corresponding author.
