# Supplementary material for: CRISPR/Cas9‐mediated editing of Bs5 and Bs5L in tomato leads to resistance against Xanthomonas
Source: Plant Biotechnol J. 2024 Jul 12;22(10):2785–7. doi: 10.1111/pbi.14404 (PMC11536453; doi:10.1111/pbi.14404)
Supplement: Supplementary file 1 — Appendix S1 Materials and Methods. [file PBI-22-2785-s002.docx]

**Materials and Methods**

**Identification of Bs5 and Bs5L in tomato**

We identified SlBs5 (Solyc09g098310.3.1) and SlBs5L (Solyc09g098300.3.1) from chromosome 9 of *Solanum lycopersicum* by searching for homologs of CaBs5 and CaBs5L from *Capsicum annuum* with BLASTP v2.9.0+ (Camacho *et al.*, 2009; Hosmani *et al.*, 2019; Hulse-Kemp *et al.*, 2018). The gene order and synteny between tomato and pepper genomes were used to assign the orthology of Bs5 and Bs5L.

**Cas9-mediated inactivation of *SlBs5* and *SlBs5L***

One single-guide RNA (sgRNA) was used to simultaneously target exon 2 of *SlBs5* and *SlBs5L*. The sgRNA sequence was cloned into a pENTR/D-TOPO-based entry plasmid containing the *Arabidopsis* U6-26 promoter to drive guide RNA (gRNA) expression and an enhanced double 35S promoter driving Cas9 expression. A gateway LR reaction (Thermo Fisher Scientific) was used to move the gRNA and Cas9 cassette into a pPZP200-based binary vector. Using the *Agrobacterium tumefaciens* co-cultivation method, the binary construct was transformed into the Fla. 8000 variety at the Innovative Genomics Institute Transformation Core Facility. Kanamycin-resistant plants were genotyped, and the selected mutants were self-pollinated for subsequent experiments.

**Bacterial and Plant material**

For all experiments, the wild type plants refer to the variety Fla. 8000 susceptible to *Xanthomonas spp.* Wild type and mutant plants were grown on soil (Miracle-Gro Supersoil Potting Soil) in a growth chamber at 25°C under a 16-h light/8-h dark photoperiod and 50% relative humidity. Experiments were performed with six-week-old plants. *Xanthomonas perforans (Xp) GEV485*, *Xanthomonas perforans (Xp) 4B*, *Xanthomonas gardneri* *(Xg) 153*, *Xanthomonas euvesicatoria (Xe) 85-10*, and *Pseudomonas syringae* pv. tomato R2 (*Pst*) were used for plant inoculation. *A. tumefaciens* strains LBA4404 and AGL1 were used for tomato transformation.

**Pathogen assays**

For pathogen assays, *Xanthomonas* bacterial cultures were grown in NYG (0.5% peptone, 0.3% yeast extract, and 2% glycerol) with 100 μg/ml rifampicin for 18 h at 28°C on a shaker at 180 rpm. After centrifugation at 4,000g for 15 minutes, cells were washed once with 10 mM MgCl_2_, and diluted to OD_600_=0.0003 for syringe infiltrated pathogen assays and OD_600_=0.003 for dip inoculation symptom assays.

For bacterial growth assays, bacterial suspension (OD_600_=0.0003, 10 mM MgCl_2_) was infiltrated into fully expanded leaves, and plants were grown over a course of up to several days. Leaf punches were collected, homogenized and then serially diluted. For quantification of bacterial populations, serial dilutions of leaf homogenates were plated onto NYGA (0.5% peptone, 0.3% yeast extract, 2% glycerol, and 1.5% agar) with 100 μg/ml rifampicin and 50 μg/ml cycloheximide. After incubation at 28°C for 4 to 5 days, typical colonies of *Xanthomonas spp* were counted, and the bacterial population on each plant was estimated.

In dip inoculation assay, three leaflets from each plant were dipped into the bacterial suspension amended with 10 mM MgCl_2_ and 0.02% Silwet L-77. Infected plants were grown over 14-21 days at 25°C until symptoms developed.

**Field trial assays**

Field trials were performed at the Gulf Coast Research and Education Center (GCREC) in Balm, Florida. Experimental plots were arranged in single rows of 10 plants each, following a randomized complete block design (RCBD) with four or five replicates. Four-week-old tomato seedlings were transplanted into raised beds covered with reflective polyethylene mulch, and irrigation was applied through drip tape beneath the plastic mulch. Throughout the growing season, plants were grown with recommended fertilizers and pest management programs, excluding the use of any bactericides or activators of systemic acquired resistance. Approximately 2 months after sowing, plants were inoculated with a two-isolate cocktail of *X. perforans* race T4 (10^6^ CFU per ml of each of strains GEV872 and GEV1001).

Bacterial spot disease severity was evaluated one month after inoculation using the Horsfall-Barratt rating scale. Vine-ripened fruits were harvested two times from eight plants per plot on separate dates, approximately one- and 1.5-months post-inoculation. Fruits were weighed and graded according to USDA standards (51.1859 of the US Standards for Grades of Fresh Tomatoes). According to the USDA specifications, only medium, large and extra-large fruits (i.e. sizes 6x7, 6x6 and 5x6, respectively) were considered for total marketable yield calculation, excluding unmarketable small fruits.

**Reference**

Camacho, C., Coulouris, G., Avagyan, V., Ma, N., Papadopoulos, J., Bealer, K., and Madden, T.L. (2009) *BLAST+: architecture and applications*. *BMC Bioinformatics*, **10**, 421.

Hosmani, P.S., Flores-Gonzalez, M., Van De Geest, H., Maumus, F., Bakker, L.V., Schijlen, E., et al. (2019) *An improved de novo assembly and annotation of the tomato reference genome using single-molecule sequencing, Hi-C proximity ligation and optical maps*. Genomics.

Hulse-Kemp, A.M., Maheshwari, S., Stoffel, K., Hill, T.A., Jaffe, D., Williams, S.R., et al. (2018) *Reference quality assembly of the 3.5-Gb genome of Capsicum annuum from a single linked-read library*. *Hortic. Res.*, **5**, 4.
