# Supplementary material for: CRISPR/Cas9‐mediated editing of Bs5 and Bs5L in tomato leads to resistance against Xanthomonas
Source: Plant Biotechnol J. 2024 Jul 12;22(10):2785–7. doi: 10.1111/pbi.14404 (PMC11536453; doi:10.1111/pbi.14404)
Supplement: Supplementary file 2 — Figure S1 Qualitative evaluation of disease symptoms with a dip inoculation assay. Figure S2 Quantitative evaluation of bacterial growth after inoculation. Figure S3 Height comparison between wild type and mutant plants. Figure S4 The disease symptoms on wild type and Slbs5‐1 plant leaves in the field trials. Figure S5 Fruit yields of wild type and Slbs5‐1 plants in the Fall 2023 field trial. [file PBI-22-2785-s001.docx]

Wild type *Slbs5*

*X. gardneri* 153

*X. perforans* 4B


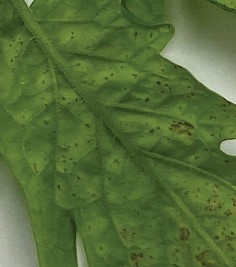

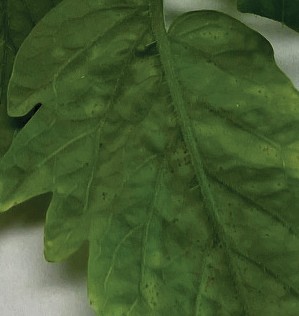

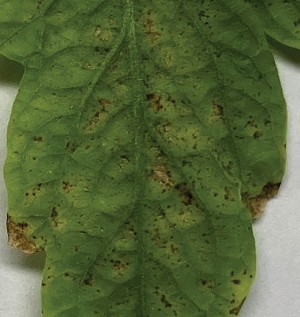

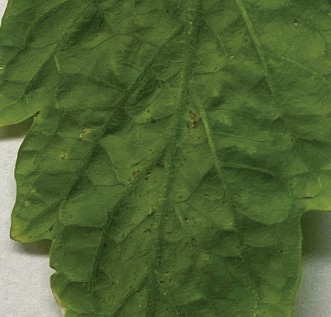

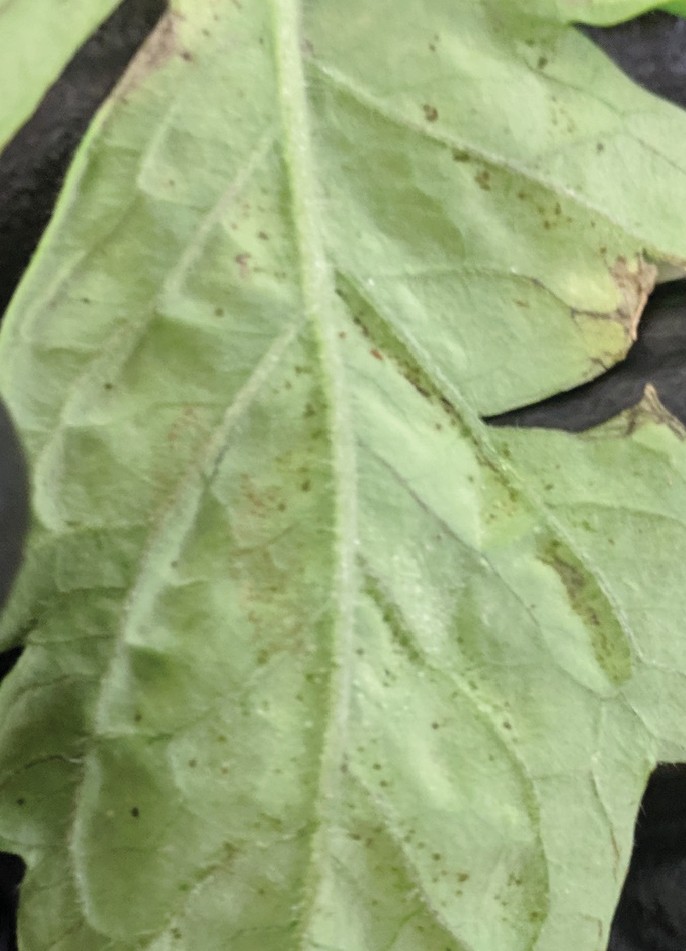

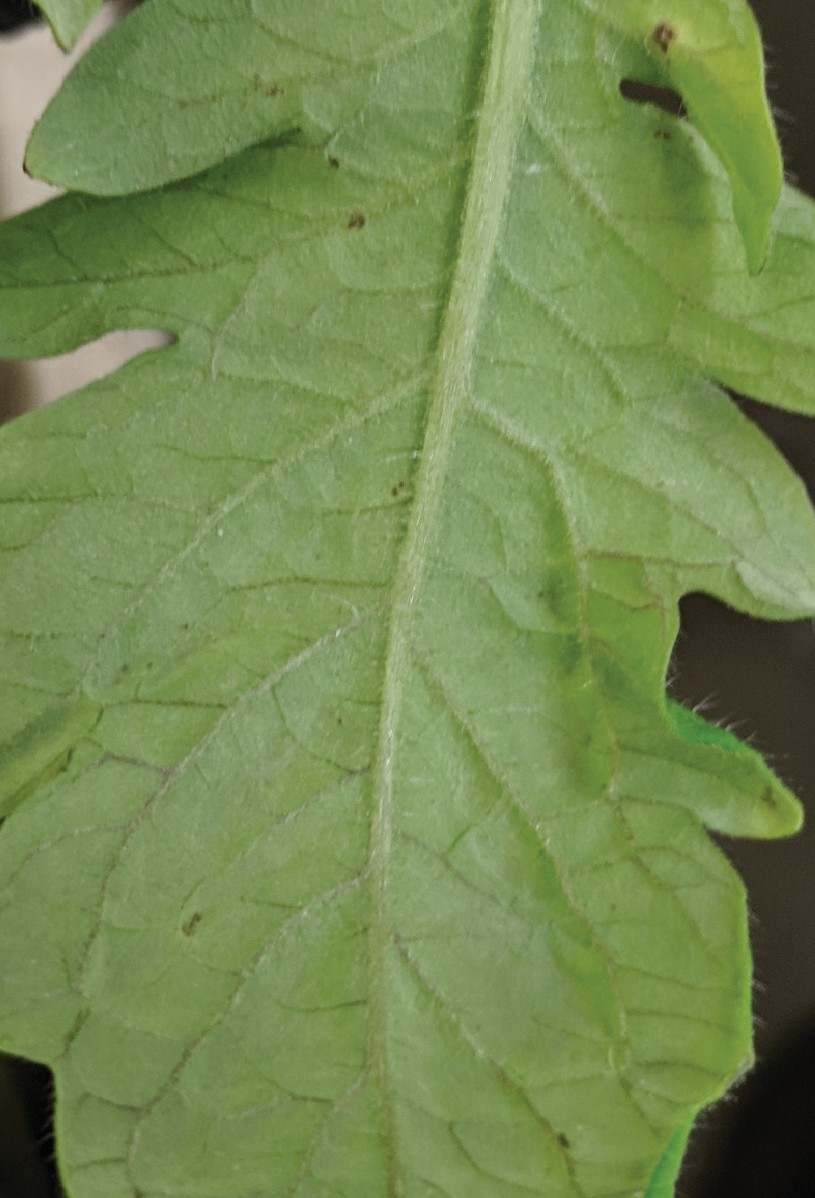


# Figure S1. Qualitative evaluation of disease symptoms with a dip inoculation assay

*X. euvesicatoria* 85-10

The wild type, *Slbs5* plants were dip-inoculated with *Xanthomonas perforans* 4B, *Xanthomonas gardneri* 153, and *Xanthomonas euvesicatoria* 85-10. The phenotypes were recorded at 21 days post inoculation, and the representative phenotypes are presented.

9


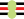

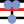

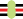

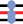


**

***

***

Wild type

*Slbs5-2*

8

Log10(CFU/cm²)

7

6

5

6 dpi

+*X. perforans GEV485*

6 dpi

+*X. perforans 4B*

6 dpi

+*X. gardneri*

# Figure S2. Quantitative evaluation of bacterial growth after inoculation

The wild-type and *Slbs5-2* plants were inoculated with *Xanthomonas perforans* GEV485 and 4B as well as *Xanthomonas gardneri* 153. The bacterial populations were recorded at six days post infiltrations (dpi). Statistical significance was determined with two-tailed pairwise t-tests with the Benjamini-Hochberg procedure applied to correct for multiple testing (** P < 0.01; *** P < 0.001).

# a


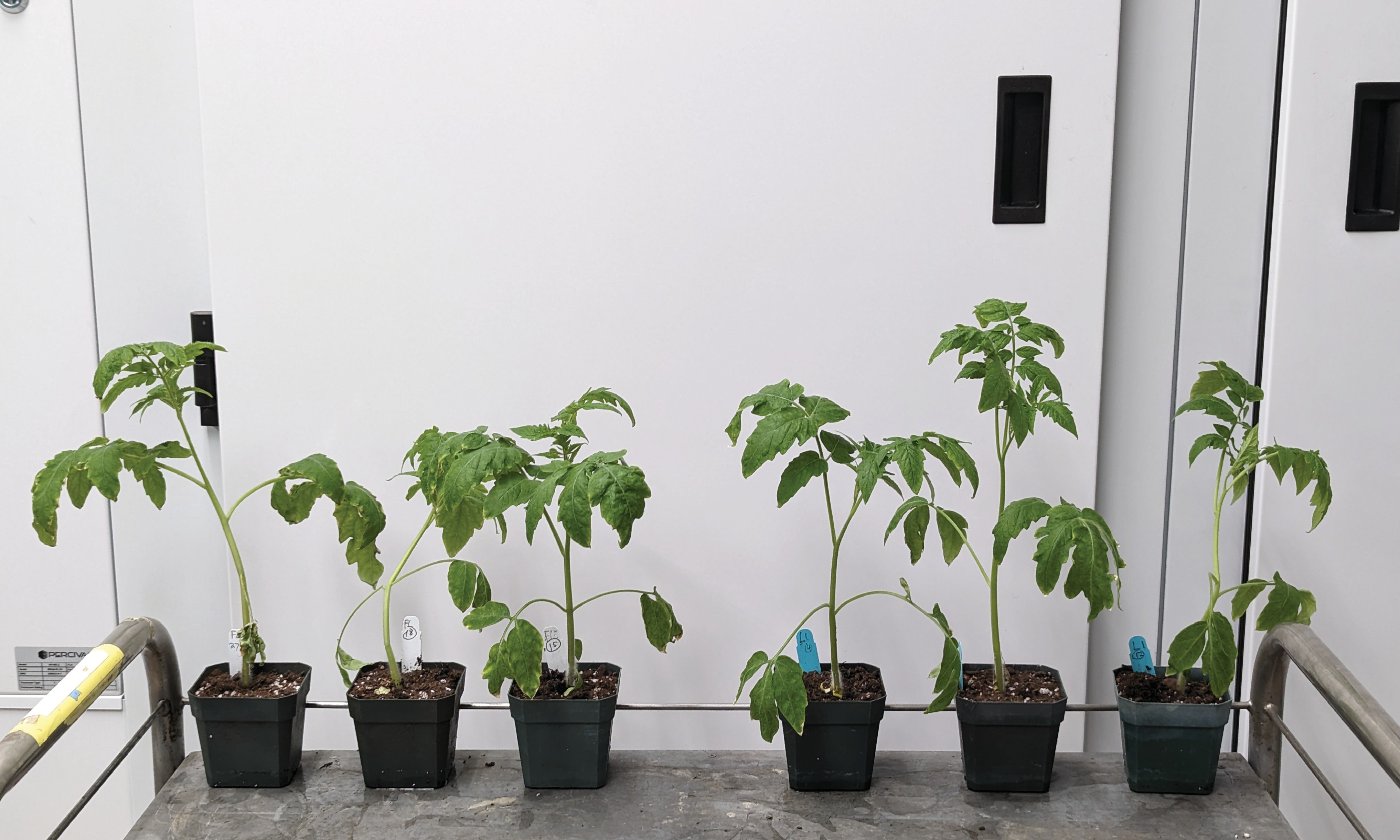


Wild type

# b

*Slbs5-1*


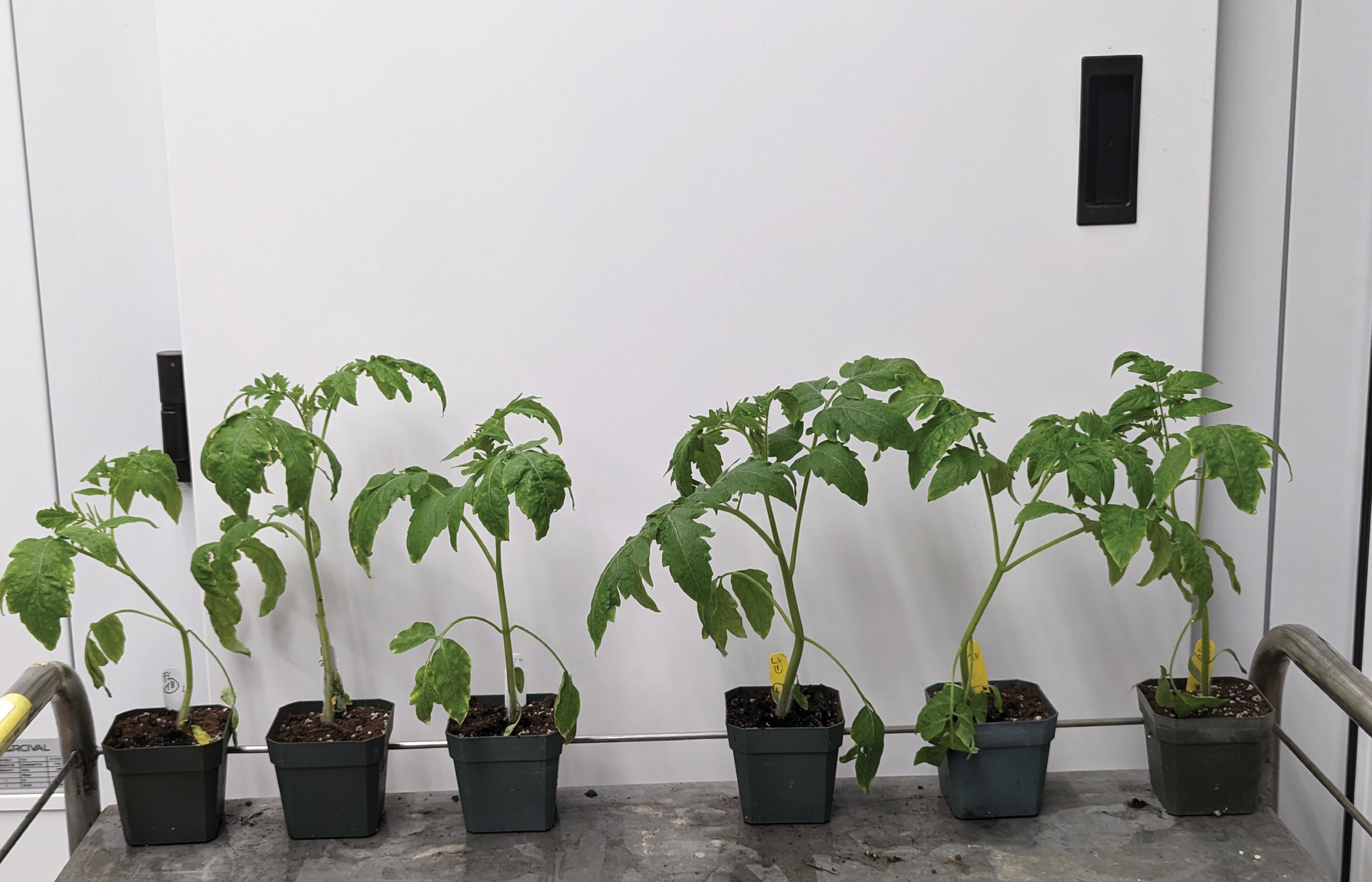


Wild type *Slbs5-2*

# Figure S3. Height comparison between wild type and mutant plants

Three represenative plants of wild type, (a) *Slbs5-1* and (b) *Slbs5-2* grown in the growth chambers for size comparisons. These photos were taken 24 days post transplanting.

Diseased leaf area (%)

0 1 3 6 12 25 50 75 100

100

2018 2019 2023

Spring Fall Spring Fall Fall

80

Examined leaf (%)

60

40

20

0

# Figure S4. The disease symptoms on wild type and *Slbs5-1* plant leaves in the field trials

The area of leaves impacted by bacterial spot disease was examined for each leaf, and this measurement was catego- rized based on the given scale.

2023

Fall

60

n.s.

50

Marketable yield (t/ha)

40

30

20

10

0

# Figure S5. Fruit yields of wild type and Slbs5-1 plants in the Fall 2023 field trial

Medium, large, and extra-large marketable fruits were harvested at the end of a field trial in Fall 2023 from wild type and *Slbs5-1* plants, and fruit quantities were measured. Two-tailed t-tests were performed with the Benjamini-Hochberg procedure applied to correct for multiple testing (n.s. P ≥ 0.05).
